# Nurses' bereavement experiences of a deceased colleague due to COVID‐19: A phenomenological study

**DOI:** 10.1002/nop2.1976

**Published:** 2023-08-22

**Authors:** Fatemeh Najafi, Leila Mardanian Dehkordi, Sajad Khodayari, Molouk Jaafarpour, Alireza Nikbakht Nasrabadi

**Affiliations:** ^1^ Department of Nursing, Faculty of Nursing and Midwifery Ilam University of Medical Sciences Ilam Iran; ^2^ Nursing and Midwifery Care Research Center Isfahan University of Medical Sciences Isfahan Iran; ^3^ Department of Critical Care Nursing, School of Nursing and Midwifery Tehran University of Medical Sciences Tehran Iran; ^4^ Department of Midwifery, Faculty of Nursing and Midwifery Ilam University of Medical Sciences Ilam Iran; ^5^ Department of Medical Surgical Nursing, School of Nursing and Midwifery Tehran University of Medical Sciences Tehran Iran

**Keywords:** bereavement experience, COVID‐19, phenomenology

## Abstract

**Aim:**

Healthcare workers have little time to mourn due to the intensification of the COVID‐19 pandemic. Although grief is a normal part of life and death, the circumstances surrounding the death can affect the grieving process. So far, the nurses' experience in mourn for a deceased colleague in the COVID‐19 pandemic has not been determined. Identifying these experiences can provide opportunities to formulate appropriate strategies to functionally adapt to death and promote mental health and well‐being during this crisis. This study aimed to understand the nurses' experiences in mourning for a deceased colleague due to COVID‐19.

**Design:**

This was an interpretive phenomenological study.

**Method:**

Participants included 10 nurses with the bereavement experience following the death of a colleague due to COVID‐19, who were selected through purposive sampling, and the data were collected through in‐depth and semi‐structured interviews and analysed using Diekelmann et al.'s (1989) approach.

**Result**s**:**

The nurses' bereavement experiences were in the form of eight themes: disbelief and amazement, acceptance with grief, lasting sadness, unsung laments, bringing back memories, impulse to leave the service, a professional myth and holy death. For nurses, mourning for the death of a colleague due to COVID‐19 is like a lasting sadness that begins with disbelief and amazement and changes to acceptance with sadness. From the fellow nurses' point of view, this type of death was perceived as a holy death, which along with countless unsung laments and memories brought to us the association of a professional legend, and that such a fate would be inevitable for us as well, it was a push to leave the service.

**Public Contribution:**

Crisis managers and policymakers need to add protocols and training programs for resilience skills and healthy mourning.

## 1 INTRODUCTION

The COVID‐19 pandemic puts healthcare professionals around the world in an unprecedented situation, forcing them to make impossible decisions and work under extreme pressure (Greenberg et al., 2020). In March 2020, Italy reported that over 2600 healthcare workers were infected with the virus (Ehrlich et al., 2020). WHO estimates that between 80,000 and 180,000 health workers may have died from COVID‐19 between January 2020 and May 2021(WHO, 2021). 300 healthcare personnel in Iran died due to COVID‐19 within 18 months (Gharebaghi et al., 2021).

The death of a healthcare worker can be disappointing. The common element in death for everyone might be the process of grief and mourning in their various forms (Rotter, 2000). For healthcare workers on the front lines of COVID‐19, the death of a colleague can be challenging. This is not only the loss of a neighbour and a friend, but may represent the loss of another fighter in this crisis. Colleagues may share training, roles and experiences. As a result, a colleague's death during COVID‐19 may lead to increased fears for personal, other colleagues' and family members' safety. With the occurrence of death, the physical, mental and social consequences and the isolation due to social distance may affect the potential of complex grief and mourning (Wallace et al., 2020).

Uncomplicated grief includes multiple emotional, cognitive, physical, and behavioural responses that are common reactions after loss (Wallace et al., 2020). Kubler‐Ross (1969) defines the grieving process as going through five stages of grief: denial (denial of loss or illness), anger (at loss or illness towards people or God), bargaining (whether there is another way?), depression and acceptance (Kubler‐Ross, 1969, p. 10). The grieving process involves going through steps to come to terms with or accept the death. For front‐line healthcare workers, the dramatic increase in mortality rate is very worrying. Due to the severity of the situation, they have little time for mourning (Zhai & Du, 2020).

While grief is a normal part of life and death, the circumstances surrounding the death can affect the grieving process. Some new challenges of grieving over a colleague's death may be related to the limitations of social distancing. All societies have a death system with the main functions of warning and prediction, prevention, care of the dying, burial of the dead, social consolidation after death, making sense of death. In times of outbreak of infectious diseases such as COVID‐19, this system along with the cultural and religious rituals, which help people overcome grief can be destroyed. Funerals may be cancelled or attendees may severely be restricted. Individuals may be limited and unable to physically contact with family, friends and other associates; this limitation lead to greater feelings of isolation. Isolation from others may cause tension and dissatisfaction (Moore et al., 2020).

Sociocultural ties and strong emotional relationships with friends and acquaintances are important in Iranian culture, and the death of acquaintances and colleagues deeply affects people's spirit. In Iran, it is customary that when a colleague dies, other colleagues attend the funeral and walk to a mosque or the deceased person's home to honour him/her, express sympathy to the bereaved family, alleviate their own grief, and use the opportunity to say farewell (Shoraka et al., 2022). For most Iranians, mourning is a major emotional and social moment, and it is believed that the details of elaborate religious rituals affect the passage of the deceased from this world to the next world. Iranians even organize a spiritual and religious memorial ceremony for the deceased colleague at work and mourn the loved one by reading the Qur'an, reciting surah Al‐Fatiha, and recalling memories of the deceased—all of which can have comforting effects (Bayatrizi et al., 2021). During the COVID‐19 pandemic, much like in other countries, new policies and guidelines were implemented in Iran regarding the management of bodies, funerals, burials and attendance of customary ceremonies, and under the conditions, all psychological and social support were taken away from mourners as they found themselves alone (Shoraka et al., 2022).

If grief is not adequately addressed by healthcare workers, cumulative losses can impair individual, team and organizational effectiveness. Strong emotions following loss are normal reactions to grief. The intensity and frequency of these feelings may intensify in stressful conditions. In response to grief, some will try to move forward and may choose longer hours and more frequent shifts. Although this may be effective in the short term, it will most likely cause burnout and loss of effectiveness in the long term (Wallace et al., 2020). A colleague's death sets the team on a path that transforms people and changes the group dynamics. While the team members are informed of their colleague's death, the team will never act in the same way as they fight together to discover a new norm (McGettigan et al., 2020). Vachon has emphasized the importance of self‐care and the need to identify and deal with stressful factors related to work for nurses (Vachon, 2001). The mourning process should be recognized by nursing and health management as a necessity not only for physical but also for mental and spiritual health (Ehrlich et al., 2020).

Literature of reviews showed some studies conducted on the bereavement experience of the general population and the relatives of deceased COVID‐19 patients (Mortazavi et al., 2021; Shoraka et al., 2022). In many studies, the effects of loss and bereavement have been examined as a part of more general studies of stress or “burnout” experienced by care providers by reason of moribund people (Kostka et al., 2021; Lobb et al., 2010). The impact of bereavement on the treatment team was similar; however, the authors could not find any studies that had examined the bereavement experience of nurses following a colleague's death who had passed due to COVID‐19.

The COVID‐19 pandemic has disrupted normal grief experiences, and it is necessary to reform approaches to grief support. If these challenges are not recognized on time and no solution is offered for their management, many other problems may arise, leading to a major public health concerns. It is important to understand how current circumstances can set the stage for bereavement. Gaining a deep understanding of the experience and encounter with grief for a deceased colleague offers insights into supporting bereaved people, identifying their psychological needs, providing proper services and helping reduce the mental harms caused by not expressing grief during the pandemic. It also helps identify proper strategies to increase health and social support programs in the future. Quantitative research cannot understand how nurses experience or what their views are, and phenomenology is a sufficiently enough approach to search deeply into personal meanings and lived experiences and ultimately to deeply understand a phenomenon including communication, expectations, attitudes and beliefs. This qualitative study aims to understand the bereavement experience of nurses following deceased colleagues.

## 2 MATERIALS AND METHODS

This is a phenomenological interpretative study, aiming to understand and analyse the nurses' experiences regarding the phenomenon of mourning for a deceased colleague due to COVID‐19. This study was conducted from April to December 2020 using an interpretive phenomenological approach to explore and understand the lived bereavement experiences of nurses following the death of a colleague from COVID‐19. The goals of this interpretive phenomenological study were to gain a deep understanding of the bereavement experience from the perspective of nurses who had lost their colleagues to COVID‐19 and access the underlying layers of this experience. The study environment was the affiliated hospitals of Tehran University of Medical Sciences. The researcher approved the study plan and obtained approval from the Faculty of Nursing and Midwifery before collecting and analysing the data. Participants were selected from nurses with different educational backgrounds and professional experience working in the clinical departments of the hospital. The study objective was clear to nurses, and after agreeing to participate in the study, the informed consent forms were obtained. They were asked to specify a time to conduct the interview. Sampling was done in a purposive method. In order to achieve maximum diversity, they were selected from different backgrounds, records and conditions in terms of age, sex, wards and work experience. The inclusion criteria were being a nurse, having bereavement experience of one colleague who had passed due to COVID‐19 in the last 3 months, having worked with the deceased nurse in the same ward and having shared a work shift with the deceased person over the last 3 months. If the participants recalled bitter memories that made them mentally unable to continue the interview, they were excluded from the study.

### 2.1 Participants

The participants were 10 nurses working in different wards (6 women and 4 men). Samples were selected through purposive sampling from among the nurses of affiliated hospitals of … University of Medical Sciences who were willing to participate in this study.

### 2.2 Data collection

Data were collected through in person interviews and in some cases through WhatsApp voices and messages in an in‐depth and semi‐structured manner. Interviews were conducted by a researcher familiar with the manner of conducting in‐depth interviews. The number of sessions and duration of each interview were different according to the conditions of each participant and depended on various factors including the time and people's willing. The average time of the interviews varied from 30 to 45 min and each participant was interviewed once. Before starting the interview, a preliminary interview guide was prepared to help the researcher ask more questions to dig into the desired area. In the interviews, basic questions like: “What comes to your mind when we say mourning for a deceased colleague due to COVID‐19? What is it like to mourn for a deceased colleague due to COVID?”, “How do you feel when a colleague dies due to COVID‐19?” and “Can you give me an example”, were raised, then deep exploratory questions such as: “Can you explain more or what do you mean when you say… and how…? Have you had a similar experience? and what do you mean by it?” were asked and at the end the participants were asked to comment if there was anything left to say.

Upon the participants' permission, the interviews were digitally recorded and then transcribed verbatim and written down. MAXQDA‐10 was used for better data management. Data collection and analysis were done simultaneously. For example, data analysis began with the first interview. Finally, by obtaining qualified participants, data collection and analysis were performed simultaneously and continued until saturation within 8 months. Interviews continued until we reached depth, richness, abstraction.

### 2.3 Data analysis

The data were analysed using Diekelmann et al.' approach (1989), a seven‐step process based on Heidegger's phenomenology, with a team approach. Each interview was first transcribed verbatim and reviewed one or more times to obtain an overall understanding of the text. For each interview text, an interpretative summary was written by the researcher and an attempt was made to understand and extract the hidden meanings in it. The team members exchanged their ideas about extracting topics and themes, and as the interviews continued, the previous topics became clearer and evolved, and sometimes new topics emerged. This was done through discussions with team members. By finding new topics, patterns and interpretations were formed, and explicit and implicit concepts were extracted from the interview texts. These implications were not just the simple statements of the participants, but included the atmosphere of the interview and the way the participants answered the questions.

To clarify, categorize and eliminate contradictions in interpretation, we returned to the texts again and again. The purpose of data analysis was to extract concepts and themes. Themes were a set of general features showing the main meaning of concepts, similarities and differences. At each stage, with the progress of the work, by combining interpretive summaries, combined and general analysis was formed so that the resulting themes and subthemes were related in the best possible way.

## 3 TRUSTWORTHINESS

The accuracy of the study is one of the important issues that should be considered in all stages of qualitative research, because it enables the reader to understand the events, effects and actions of the researcher (Elo et al., 2014). In this study, appropriate context and information sources, qualified participants, close and long‐term and continuous interaction with the participants, specifying and clarifying the work steps, the manner of the processes (to make it easier for others to understand), presenting the draft version from the themes along with selected excerpts from the interviews to the members of the analysis team, adopting a team approach using collective team discussion, performing analytical comparisons, and cross‐referencing the raw data ensured the accuracy and validity.

## 4 ETHICAL CONSIDERATIONS

This study was approved by the Ethics Committee of the Faculty of Nursing and Midwifery of … University of Medical Sciences (…). The purpose and method of the study were explained to the subjects participating in the study and they were told that they could withdraw from the study at any time without penalty or damages. Verbal and written informed consent were also taken from them before participating in the study and recording the interviews. They were also assured of the confidentiality of their information and responses.

## 5 RESULTS

A qualitative phenomenology study with an interpretive approach has been carried out to understand the nurses' bereavement experience following a deceased colleague due to COVID‐19. Data were obtained from 10 in‐person interviews with participants, which continued until data saturation was reached. The average age of participants was 37.4 and 6 of them were women and 4 were men. They also had bachelor's and master's degrees. The participants in this study expressed their opinions and experiences regarding the mourning for a deceased colleague due to COVID‐19 (Table 1).

**TABLE 1:** Demographic characteristics.

Participant	Sex	Age	Education	Work experience (year)	Workplace	Marital status
1	Male	49	Bachelor of Nursing	29	Medical	Married
2	Male	30	Bachelor of Nursing	12	Emergency	Single
3	Female	28	Bachelor of Nursing	4	Emergency	Married
4	Male	25	Bachelor of Nursing	3	ICU	Single
5	Female	49	Bachelor of Nursing	21	Haematology	Married
6	Female	40	Bachelor of Nursing	11	ICU	Married
7	Female	35	Master of Nursing	10	Medical	Single
8	Male	31	Master of Nursing	9	Emergency	Single
9	Female	48	Bachelor of Nursing	24	Emergency	Married
10	Female	39	Bachelor of Nursing	14	ICU	Married

According to the detailed and in‐depth description of the participants and data analysis, eight themes were extracted including: disbelief and amazement, acceptance with grief, lasting sadness, unsung laments, bringing back memories, impulse to leave the service, a professional myth and holy death, which are explained in detail (Table 2).

**TABLE 2:** Themes and subthemes.

Theme	Subtheme	Quotations
Disbelief and amazement	Amusement	“It was more like a shock. At first, no one could believe that our colleague died, because we did not believe his death”
	Disbelief	“I often wait for him to come to the shift, I cannot accept that he has left us and is no longer with us”
Acceptance with grief	Forced acceptance	“Even if you do not want to accept it, the death has happened and unfortunately you have to accept it”
	Gradual acceptance	“I put a table in the hospital lobby, I had ordered the pictures and the black cloth the day before, we spread the Ziarat‐e‐Ashura (a prayer), we donated water and chocolate with a black cover, and I wanted to alleviate the personnel sufferings”
Lasting sadness	Physical suffering	“The news of his death brought tears to my eyes, I cried uncontrollably”
	Mental suffering	“I was very sad to lose this friend and colleague and I am still sad”
Unsung laments	Regrets for not participating in the funeral ceremonies	“What is a tradition in our society and in our ceremonies is that people can reach the corpse and vent themselves and this helps them to reduce their sadness and grief a little, well, in this situation, since he died due to COVID‐19, this situation is not available, and this grudge, sadness and regret will remain with us …”
	Self‐destruction	“I was upset that why we could not do something and why we found out so late …”
Bringing back memories	Remembering back memories	“I reminded others of the memories in the virtual space and expressed the sadness that our deceased colleague experienced in the past”
	Keeping memories alive	“Bringing back memories never ends and I want to keep his memories alive in my mind forever. His memories are constantly reviewed”
An impulse to leave service	Waiting for a similar fate	“What made our colleague's death sadder was that, we are at the front line of treatment, and all kinds of problems threaten us”
	Job despair	“His death has affected me so much both in terms of work and mentally that I am looking for the first opportunity to write my letter of retirement and actually get rid of this job”
Professional myth	Professional union	“It feels very bad to leave my colleagues alone in this situation, we have to accept this situation to endure and unify to defeat this virus”
	Professional pride	“it is true that one's life is important to him, but serving in this situation is much more important, and the people around me, the patient and the patient's companion, look at me as a hero and this is very valuable …”
Holy death	Self‐sacrifice	“Even though he knew what was waiting for him, he served more than his duty. I am proud of this death. It gave us honour in a way”
	Oppressed death	“It is like a death that seems to have no one, very lonely, and left alone”

### 5.1 Disbelief and amazement

Participants considered their experience of mourning for a deceased colleague due to COVID‐19 as disbelief and amazement. In this category, subthemes such as amazement and disbelief were described. The majority of participants stated that they were shocked by their colleague's death due to COVID‐19 and that his/her death was unbelievable for them. “It was a really a bad feeling, it was more like a shock. At first, no one could believe that our colleague died, because we didn't believe his death, because he was so young and it happened suddenly, it was very hard for us.” (P3).

Some have also stated that they were still waiting for their colleague to return to the shift and it was hard to believe their death. A participant says: “I often wait for him to come to shift, I can't accept that he has left us and is no longer with us” (P2).

### 5.2 Acceptance with grief

Nurses have experienced the mourning for a deceased colleague due to COVID‐19 in the form of acceptance with sadness. In this theme, the subthemes of forced acceptance and gradual acceptance are described. Some participants have stated that they are used to the death and absence of colleagues due to compulsion. One of the participants stated: “Even if you don't want to accept it, the death has happened and unfortunately you have to accept it” (P4).

According to some participants, accepting the colleague's bereavement happens gradually and they will cope with it over time. In the gradual acceptance of this incident, some have pointed to measures such as holding a memorial in the hospital and workplace, empathy with the survivors and paying attention to spirituality, which has helped them gradually come to terms with this issue. One of the participants said: “I put a table in the hospital lobby with the help of my other colleagues, I had ordered the pictures and the black cloth the day before, we spread the Ziarat‐e‐Ashura (a prayer), we donated water and chocolate with a black cover, and I wanted to alleviate the personnel suffering”(P9).

Some participants have also stated that due to the nature of their jobs, they more easily accept loss. One of them expressed: “Nurses accept the loss sooner because they face the phenomenon of people's death every day, especially as I who works in a blood cancer ward” (P5). This is the manifestation of the problem, people do not seem to forget the grief and memories of their fellows so quickly. Another participant said: “I soon dealt with death, but the feeling of sadness was always there, it is true that I get along soon, but we were colleagues and I never forget the memories of me with my colleagues on the front line of COVID‐19 and the fact that they passed because of COVID‐19 is very bitter” (P4).

### 5.3 Lasting sadness

Most of the participants stated that they have experienced the phenomenon of long‐lasting grief and suffered physical and mental damage as they mourned for a deceased colleague due to COVID‐19. In this theme, subthemes such as physical and mental sufferings are described.

They have stated that after hearing the news of their colleague's death due to COVID‐19, tears flowed down their cheeks and some of them also felt anger, sadness and heart compression. One of the participants said: “The news of his death brought tears to my eyes, and when I received the news from the virtual group, I cried uncontrollably” (P6).

All the participants mentioned many things about the experience of mental suffering caused by a colleague's death due to COVID‐19. They have stated that their colleague's death was tormenting, bitter and painful for them. They felt sadness and death was heavy and difficult for them. One of the participants stated in this context: “I was and am still very sad, it was very difficult to accept the fact that I lost a friend and a colleague due to COVID‐19” (P1).

Some participants have considered a colleague's death as a member of their family. Sometimes these problems are so severe that nurses feel depressed. A participant stated: “My colleague's death was very bad and I was deeply depressed” (P7).

### 5.4 Unsung laments

The participants have explained the unsung laments of the mourning for the deceased colleague due to COVID‐19. The theme of unsung laments includes subthemes like regrets for not participating in the funeral and memorial ceremonies of colleagues and the subtheme of self‐destruction and the dominance of negative thoughts. Due to the COVID‐19 pandemic and the spread of this infectious disease, they could not perform traditional mourning and attend funeral ceremonies and visit the graves of their deceased colleagues. One of the participants stated: “What is a tradition in our society and in our ceremonies is that people can reach the corpse and vent themselves and this helps them to reduce their sadness and grief a little, well, in this situation, since he died due to COVID‐19, this situation is not available, and this grudge, sadness and regret will remain with us …”(P1).

Self‐destruction and the dominance of negative thoughts of the participants have been experienced as another example of unsung laments in the mourning for a deceased colleague. They have expressed their sadness that they could not do anything to save their deceased colleagues. One of the participants expressed in this context: “I was upset that why we could not do something and why we found out so late…” (P3). They also expressed sadness and regret about the death of their young colleagues and their failure in their personal lives. One of the participants in this context said: “Two days before his death, I saw him and we talked a lot about retirement and what he wanted to do, it's a pity that all his hard work was in vain” (P1).

### 5.5 Bringing back memories

Another experience of the participants in a colleague bereavement due to COVID‐19 was to bring back memories, which was explained by all the participants. This theme includes the subthemes of remembering memories and keeping memories alive. They mourned for their colleagues by reviewing past memories and watching photos and videos of deceased colleagues and reviewing the memories of deceased people as a group in virtual space. One of the participants stated: “I reminded others of the memories in the virtual space and expressed the sadness that our deceased colleague experienced in the past after the death of a fourteen‐year‐old teenager” (P5). Most of the participants missed the deceased person when bringing back memories.

Some participants talked about keeping their memories alive and stated that the memory of their deceased colleagues will not be forgotten. “Bringing back memories never ends and I want to keep his memories alive in my mind forever. He was very committed and virtuous, that's why I will never forget him. He is always in my mind, and his memories are constantly reviewed, many of them are good sweet and funny memories cause this grudge and sadness” (P8).

They understood their bereavement experience by talking about the deceased person and talking to the deceased colleague in their mind. One of the participants stated: “I recalled his memories more, we had a lot of memories together, I kept remembering those memories and sometimes I would talk to him and I would laugh and I would get sad and want to cry…” (P1).

### 5.6 An impulse to leave service

Some participants talk about the professional challenges and the urge to leave the service that they experienced during the mourning process for a deceased colleague due to COVID‐19. Most of them were in two minds after the death of their colleagues, and some have thought of leaving the service. In this finding, the subthemes including: waiting for a similar fate and job despair have been described. Most of the participants were expecting a similar fate for themselves due to their colleagues' death. More than other times, they have seen themselves exposed to danger and job threats and even death, and some have even stated that their death is certain. One of the participants stated: “What made our colleague's death even sadder was the fact that unfortunately we are at the front line of treatment, and all kinds of problems threaten us” (P1). The majority of them have mentioned their fear of infecting themselves, their colleagues and their families. A participant stated: “The moment someone dies from a disease like COVID‐19, a disease that we are all dealing with, we are working with the patients. When it happens, it means he was the first person, then it's my turn. Well, there is definitely a stress behind it and we would think I would be the next to die…” (P2) “I felt fear and stress. I was afraid of getting sick and lest my other friends and I get infected again and God forbid bad things happen” (P10).

Some participants in the mourning process for a deceased colleague due to COVID‐19 have experienced despair towards their jobs and careers. They have become frustrated, helpless, unmotivated and unwilling to do their work. “It was disappointing and discouraging to me that no one would care about us, neither the authorities nor the people, we were telling people to stay at home and no one would listen, the authorities were not paying attention to our material rights and equipment needs. It was our duty, but when I felt that our lives were in danger and the expert forces were dying, I came to the conclusion that I would go out of COVID‐19 wards and work in non‐COVID wards” (P4). Some said they were plagued with anxiety and distress at work and were considering quitting their jobs. “His death affected me both professionally and mentally and I am looking for the first opportunity to actually quit this job by writing a letter of resignation” (P1).

### 5.7 Professional myth

Some addressed another aspect of their bereavement experiences for a deceased colleague in relation to their careers. They have described their experience of mourning for a deceased colleague as a professional myth. Subthemes such as professional union and professional pride are placed in this theme. They noted that nurses' solidarity, empathy, and acceptance of occupational risks could overcome the virus. One of the participants in this field stated: “It feels very bad to leave my colleagues alone in this situation, we have to accept this situation to endure and unify to defeat this virus” (P10).

It seems that with a colleague's death due to this dangerous and deadly disease that has become a pandemic all over the world and the treatment team and nurses who have the most exposure and effective care in caring for patients, it creates a positive attitude and a sense of pride towards the profession in them. They state that they are referred to as heroes and that they are proud of the strength of their field of study. One of the participants stated: “When I heard about it, I couldn't work, then I came to the conclusion that it was true that one's life is important to him, but serving in this situation is much more important, and the people around me, the patient and the patient's companion, look at me as a hero and this is very valuable…”(P2).

### 5.8 Holy death

Some participants mentioned the bereavement experience of a deceased colleague due to COVID‐19 as an encounter with the holy death. In this finding, according to the participants, the subthemes of self‐sacrifice and oppressed death are examples of holy deaths. These participants have considered martyrdom and scarify their lives in the way of service and death by sincerely serving by their deceased colleagues due to COVID‐19 as martyrdom. One of the participants stated: “My colleague will always be alive and immortal because she was the savior of the tired and suffering body of many people and he lost his life in the way of serving the people” (P5). According to some participants, the deceased colleagues have in some way shielded themselves and acted beyond their duty. One of the participants stated: “Even though he knew what was waiting for him, he served more than his duty. I know it made the medical staff proud and I am proud of this death. It gave us honor in a way” (P4).

Some participants in the mourning process for a deceased colleague due to COVID‐19 understood their death as a kind of oppressed and lonely death. They have considered and experienced the colleague bereavement due to illness as dying with oppression due to lack of equipment and in a way a painful and lonely death. Another participant stated: “It is like a death that seems to have no one, very lonely, and left alone” … (P1). Some also experience the mourning for deceased colleague due to COVID‐19 as the loss of a national capital that they were seen after their death, and somehow suffered an oppressed death. “My colleague's death is like the loss of a national capital, like the loss of a warrior” (P9).

## 6 DISCUSSION

It is well‐known that the COVID‐19 pandemic has caused grief and mourning for many people worldwide. Nurses were a group whose mental health was deeply affected by the pandemic. Nurses have been more exposed to the death and loss of loved ones, friends and colleagues compared to other groups during the pandemic. The death of a colleague had real emotional consequences and deep grief for nurses. This study examined nurses' experiences regarding a colleague bereavement which happened due to COVID‐19.

Based on the evaluated findings, main themes were extracted, that is, disbelief and amazement, acceptance with grief, lasting sadness, unsung laments, bringing back memories, impulse to leave service, a professional myth and holy death. Subsequently, for each main theme, several subthemes were found, each of which has been explained below in detail.

In this study, colleagues' death was very shocking due to the sudden onset of the COVID epidemic and the high death toll. Being shocked is the first reaction to the death of a loved one (Shoraka et al., 2022). As the participants in this study pointed out, facing the loss of a loved one may be a shocking experience. Such reactions are expected behavioural and emotional consequences. They can be described as preliminary bereavement. However, the shock of unexpected COVID‐19 deaths, coupled with self‐quarantine and limited or no access to family support, can reinforce denial and anger about death and cause long‐term unresolved grief (Maddrell, 2020). Anyone hearing about the death of their loved one or colleague would be devastated and would try to deny it. They do not accept it and try to make themselves believe it is not real and did not happen. For those whose loved ones lost their lives due to an accident, murder or suicide, disbelief prevailed in the early weeks, and anger and depression were present during most of the mourning period (Neimeyer, 2014).

Some participants were still waiting for their colleagues to return to the shift, and it was hard to believe their death. A few have stated that due to the nature of their jobs, they accept it more easily. These include Kübler Ross and Kessler's nonlinear stages of grief (denial, anger, bargaining, depression and acceptance), and such findings contradict the relevance of “one‐size‐fits‐all” models of grief about adapting to the loss. However, many people in modern society react as if death does not exist (Kubler‐Ross, 1969; Neimeyer, 2014).

Others have tried to find creative ways to seek and give comfort, and by holding memorials and spiritual ceremonies for deceased colleagues, they have been on the path of acceptance. It seems that having a farewell ceremony in the face of death, both before and after death, helps to give meaning to the reality of death and recognize the loss and acceptance (Hernández‐Fernández & Meneses‐Falcón, 2021). When bereavement is experienced, support strategies for healthcare workers include recognizing loss and expressing grief privately and, perhaps more importantly, publicly. Attending remote memorial services or bereavement support groups is appropriate. For many, small gestures or more formal rituals such as a brief and simple pause after hearing or witnessing a death, saying the names of those who have passed away, lighting a candle, and reciting poetry can help reduce the chaos of life and enhancing meaning (Rabow et al., 2021). But nothing seems to replace a real in‐person mourning service (Bayatrizi et al., 2021).

It is unrealistic to expect healthcare workers to immerse themselves in suffering daily and be unaffected by it. They work on the front lines of COVID‐19 and are saddened by the death of their patients, colleagues and loved ones (Rabow et al., 2021). Most of the participants, in mourning a colleague who died from COVID‐19, have experienced heavy and lasting losses and suffered physical and mental damage. Loss is a natural part of the human experience, but it can be extremely painful and negatively affect physical and mental health. Its symptoms include intense sadness that lasts more than 6 months after the death, separation distress, disturbing thoughts and feelings of emptiness (Selman et al., 2020). In other studies, it has been stated that the intensity and frequency of these feelings and grief may be aggravated in stressful environmental conditions and lack of preparation for death, circumstances surrounding the loss, type of death and lack of social support network, and basic social challenges which lead to complicated grief (Hernández‐Fernández & Meneses‐Falcón, 2021; Maddrell, 2020; Wallace et al., 2020). Nurses are also in stressful environmental conditions and difficult and deadly conditions of the COVID‐19 epidemic, so the severity of these complex problems is greater.

Some bereaved people experience symptoms such as depression, anxiety and functional disorders that go beyond grief reactions (Chen, 2022; Wallace et al., 2020). Complex grief reactions can potentially lead to long‐term adverse physical and mental health outcomes such as high blood pressure, heart diseases, insomnia, anxiety, depression, long‐term grief disorder, post‐traumatic stress disorder and suicidal thoughts (Brophy et al., 2021; Chen, 2022; Galvin et al., 2020; Rabow et al., 2021). In this paper, this adversity is so severe that nurses feel depressed.

Participants have been experiencing a form of unfinished mourning during the COVID‐19 pandemic since they are deprived of partaking in the rituals that normally occur after someone's death. They are thus left alone to cope with their grief in an unknown way (Bayatrizi et al., 2021). Clinically, “grief” includes the psychobiological responses to bereavement and is expressed through the spaces and performances of mourning. Funerals and memorials, symbolic performances embedded in cultural practices, provide a meaningful and affirming experience for the bereaved (Chen, 2022). In the Iranian culture, holding a grand funeral and attending it are critical for the deceased's friends, acquaintances and colleagues and help them pass through the stages of mourning (Shoraka et al., 2022). Grieving people need a final goodbye that allows them to close one stage and start another stage (Hernández‐Fernández & Meneses‐Falcón, 2021). When people do not have a chance to participate in mourning ceremonies and say goodbye to the deceased, they may experience more severe psychological symptoms such as anxiety, depression, anger, feeling guilt and complicated grief (Albuquerque et al., 2021; Carr et al., 2020; Chen, 2022). Calling the family and other colleagues of the deceased on the phone to express sympathy, reduce the psychological burden and console one's grief are potentially effective methods of dealing with this unexpressed mourning.

Self‐destruction and overwhelming negative thoughts of the participants have been experienced as another example in mourning for a deceased colleague. In line with the present study, McCallum et al. (2021) have stated that many nurses have cared for their colleagues who have become ill with COVID‐19. Caring for your colleagues and friends, especially with real emotional consequences, accompanied by feelings of guilt—“Why can't we save these colleagues who have worked so hard to save others?”—is accompanied by great sadness when a colleague dies (Chen, 2022; McCallum et al., 2021). Such words indicate the bereaved's tendency to seek recourse to bargaining as a defence mechanism. However, if such negative self‐talks continue, it prevents the person from accepting their grief (Shoraka et al., 2022).

Another theme of mourning a deceased colleague in the current study was to bring back memories and keep their memories alive. Bereaved people usually go through certain milestones as they adapt to their grief. They can tell the story of their loved one's death to themselves and share it with others. This is characterized by longing or preoccupation with memories of the deceased, along with other evidence of grief (Goveas & Shear, 2021). Bringing back memories can sometimes strengthen part of the adjustment phase during the grieving process. One treatment model was developed by Shear et al. to both enhance adjustment to lose and promote recovery of life goals and roles. Engaging in imaginal conversations to recreate the attachment relationship with the deceased and reviewing pleasant and sad memories related to the deceased helps the bereaved to establish a more positive memory in the mind (Shear et al., 2011).

Based on the present study's findings, another theme is professional challenges and the urge to leave the service. Due to the death of their colleagues, they have been waiting for a similar fate and death for themselves, which has caused some to quit their jobs. One of the negative impacts of the death of a colleague on nurses is the psychological effects of mourning, and anxieties and concerns about being infected and its possible consequences. The death of a co‐worker during COVID‐19 may lead to increased fear for personal, other co‐workers', and family members' safety, which may affect the potential for complex grief (Wallace et al., 2020). McCallum's study is in line with the present study, which noted that caring for colleagues and friends was especially associated with real emotional consequences and with the feeling of fear—“It could happen to me” (McCallum et al., 2021). Many healthcare workers fear bringing home COVID‐19 and putting their loved ones at risk because of their profession. Healthcare workers themselves may die from COVID‐19 as members of an affected community (Giménez‐Espert et al., 2020; Rabow et al., 2021).

Another theme extracted in this study was hopelessness and despair towards their jobs. Many healthcare workers died before being protected by using adequate personal protective equipment or relocating to low‐risk areas (McCallum et al., 2021). This awareness of health inequality related to COVID‐19 has caused more discomfort and anger, anxiety, sadness, neglect, abandonment and exploitation, uncertainty, and despair in healthcare workers (Brophy et al., 2021; McCallum et al., 2021). The findings of this study are in line with it. Some have talked about frustration, lack of motivation, and unwillingness to perform care, and they were thinking of getting rid of their job, which can be caused by incompatible grief. Incompatible grief can lead to distance and emotional depression, unsympathetic, anger and burnout. This discordant grief leads nurses to leave for other professions or poor nursing care, resulting in a severe shortage of nursing staff across the country. He also mentioned that the morale of the staff and the provision of patient care could be affected. Its consequences for the hospital can lead to high turnover and reduced service and patient satisfaction, which is in line with the results of the study by Bernoulli (2005).

Some say that they are remembered as professional heroes and legends, and they are proud of the power of their field of study, and their colleagues' death has given them honour. This study's results align with the literature and previous studies. Other studies also mentioned that nurses are the main heroes in the fight against COVID‐19 because they play an important role in testing, treating, and controlling the virus. Doctors, nurses or other healthcare professionals are the true contemporary heroes who dedicate their time, life and health to help those most in need (Różyk‐Myrta et al., 2021). By being on the front lines of the fight against the COVID‐19 pandemic, nurses are impacting other healthcare workers, especially nurses who are afraid. Nurses are there to help provide safe, timely, effective and equitable healthcare, which is their legacy, privilege and honour (FirstPost, 2020; Różyk‐Myrta et al., 2021).

Some have pointed to the defeat of the virus with the unity and empathy of nurses and the responsibility and acceptance of job risks. In Brophy et al.'s study, it was mentioned by the nurses participating in the study that if the nurses do not do this work, who will do it? (Brophy et al., 2021). In the present study, one participant mentioned the same issue, which refers to their responsibility and acceptance of job risks. Healthcare workers may work long hours on the frontline of COVID‐19 without enough rest for days and weeks, showing employees empathy and unity (Rabow et al., 2021).

The participants considered martyrdom and sacrificing their lives in the way of service and death by sincerely serving deceased colleagues due to COVID‐19 as self‐sacrifice. As nurses have always been present in all scenes of care, in this era as well nurses are the stars of this epidemic worldwide. Their courage, dedication and perseverance have become very valuable (Różyk‐Myrta et al., 2021). The slogan of the heroic health of healthcare workers has made their deaths seem inevitable and sacrificial. Healthcare and social care workers carry an almost impossible burden. Their emotional‐viral burden for many is combined with professional fatigue and moral damage (Maddrell, 2020). In the analysis of Mohammad et al., it is also mentioned that the media often use the religious concepts of martyrdom to describe the selflessness and heroism of nurses in uncertain and dangerous situations (Mohammed et al., 2021). In the present study, the participants also referred to the sacrifice and martyrdom of their colleagues as holy deaths.

The participants have considered and experienced the death and bereavement of a deceased colleague due to COVID‐19 as a death of oppression due to the lack of equipment and, in a way, a death in loneliness and oppression. The quality of dying can predict complicated grief (Rabow et al., 2021). Deaths in solitude are caused by the rules of isolation of COVID‐19 to prevent the spread of the virus. Such situations may have serious and unknown psychological consequences for family members and healthcare providers, such as feelings of helplessness, hopelessness, guilt, moral distress and long‐term, complicated grief (Voultsos et al., 2022). In a study, it is stated that dying alone is an unprecedentedly unbearable and devastating experience and is not compatible with what is called a “good death” (Voultsos et al., 2022). But due to public health restrictions, patients die alone (Chochinov et al., 2020), and in this study, participants have stated that their colleagues died alone, which affected their mourning process.

A possible limitation of this study is that it may not be generalized outside of Iran because different countries and cultures have unique expectations, practices and rituals towards death. Another limitation is that some interviews were conducted through virtual and audio on WhatsApp during the peak of COVID‐19. The strength of this study is that it is one of the first studies that qualitatively examine nurses' bereavement experience for a deceased colleague due to COVID‐19. This study assures nurses that the health system respects their needs, attempts to solve their problems, and promotes their physical, mental and social health. It is suggested that future research should focus on confirming this study's results in wider medical centres. Also, future qualitative research should be conducted on the bereavement experiences of different groups of healthcare workers, how to prevent and treat complex bereavement in them during COVID‐19 and how these processes evolve.

## 7 CONCLUSION

Mourning a deceased colleague due to COVID‐19 is like a lasting sadness for hospital nurses that starts with disbelief and amazement and changes to acceptance with grief. From the point of view of fellow nurses, this was a holy death, which along with countless unsung laments and bringing back memories brought to us the association of a professional legend. With the association that such a fate would also be inevitable for us, it was a push to leave the service. This research can help policymakers and officials plan and implement measures that may reduce the adverse consequences of bereavement during the COVID‐19 pandemic. In the programs to deal with epidemics, it is important to pay attention to the physical and mental needs of healthcare workers and provide adequate support to those exposed to multiple bereavements. In anticipating future crises, crisis managers and policymakers should add protocols and training programs for resilience and healthy mourning skills, enabling proper mourning for healthcare workers both before and after the death of colleagues and relatives.

## 8 IMPLICATIONS FOR CLINICAL PRACTICE

Nurses are the first point of contact in health systems. In general, nurses' contribution to global health is certain, and investing in their quality of life benefits society. The dire trajectory of this pandemic has reinforced communities' total dependence on a skilled and flexible nursing workforce. However, the death of a colleague puts the team on a path that transforms people and changes the dynamics of the group, and the bereavement of a colleague in the long term will cause burnout and loss of effectiveness of care. It is, therefore, necessary to consider changes in practices and legislation and to design strategies that can facilitate functional adaptation to bereavement in employees while promoting mental health and well‐being during the COVID‐19 pandemic.

## AUTHOR CONTRIBUTIONS

Study concept and design and critical revision of the manuscript for important intellectual content: Fatemeh Najafi and Alireza Nikbakht Nasrabadi; collecting data: Fatemeh Najafi, Alireza Nikbakht Nasrabadi, Leila Mardanian Dehkordi and Sajad Khodayari; analysis and interpretation of data: Fatemeh Najafi, Alireza Nikbakht Nasrabadi, Leila Mardanian Dehkordi and Molouk Jaafarpour; drafting of the manuscript: Fatemeh Najafi, Alireza Nikbakht Nasrabadi and Sajad Khodayari. All authors read and approved the final manuscript.

## CONFLICT OF INTEREST STATEMENT

There was no conflict of interest between the authors.

## ETHICS STATEMENTS

This study was approved by the Ethics Committee of the Faculty of Nursing and Midwifery of Tehran University of Medical Sciences (IR.TUMS.FNM.REC.1399. 568). The purpose and method of the study were explained to the subjects participating in the study and they were told that they could withdraw from the study at any time without penalty or damages. Verbal and written informed consent was also taken from them for participation in the study and recording the interviews. They were also assured of the confidentiality of their information and responses.

## Data Availability

The data that support the findings of this study are available on request from the corresponding author. The data are not publicly available due to privacy or ethical restrictions.

## References

[nop21976-bib-0001] Albuquerque, S. , Teixeira, A. M. , & Rocha, J. C. (2021). COVID‐19 and disenfranchised grief. Frontiers in Psychiatry, 12, 638874. 10.3389/fpsyt.2021.638874 33643101 PMC7907151

[nop21976-bib-0002] Bayatrizi, Z. , Ghorbani, H. , & Taslimi Tehrani, R. (2021). Risk, mourning, politics: Toward a transnational critical conception of grief for COVID‐19 deaths in Iran. Current Sociology, 69(4), 512–528. 10.1177/00113921211007153

[nop21976-bib-0003] Brophy, J. T. , Keith, M. M. , Hurley, M. , & McArthur, J. E. (2021). Sacrificed: Ontario healthcare workers in the time of COVID‐19. NEW SOLUTIONS: A Journal of Environmental and Occupational Health Policy, 30(4), 267–281. 10.1177/1048291120974358 PMC7804366 33174768

[nop21976-bib-0004] Brunelli, T. (2005). A concept analysis: The grieving process for nurses. Nursing Forum, 40(4), 123–128. 10.1111/j.1744-6198.2005.00024.x 16371122

[nop21976-bib-0005] Carr, D. , Boerner, K. , & Moorman, S. (2020). Bereavement in the time of coronavirus: Unprecedented challenges demand novel interventions. Journal of Aging & Social Policy, 32(4–5), 425–431. 10.1080/08959420.2020.1764320 32419667

[nop21976-bib-0006] Chen, C. Y. (2022). Grieving during the COVID‐19 pandemic: In‐person and virtual “goodbye”. OMEGA‐Journal of Death and Dying, 21, 00302228221090754. 10.1177/00302228221090754 PMC9024087 35446731

[nop21976-bib-0007] Chochinov, H. M. , Bolton, J. , & Sareen, J. (2020). Death, dying, and dignity in the time of the COVID‐19 pandemic. Journal of Palliative Medicine, 23(10), 1294–1295. 10.1089/jpm.2020.0406 32639895 PMC7523014

[nop21976-bib-0039] Diekelmann N., Allen D., Tanner C. (1989). A hermeneutic analysis of the NLN criteria for the appraisal of baccalaureate programs. In The NLN criteria for appraisal of baccalaureate programs: A critical hermeneutic analysis (pp. 11–34). National League for Nursing.

[nop21976-bib-0008] Ehrlich, H. , McKenney, M. , & Elkbuli, A. (2020). Protecting our healthcare workers during the COVID‐19 pandemic. The American Journal of Emergency Medicine, 38(7), 1527. 10.1016/j.ajem.2020.04.024 32336585 PMC7162741

[nop21976-bib-0009] Elo, S. , Kääriäinen, M. , Kanste, O. , Pölkki, T. , Utriainen, K. , & Kyngäs, H. (2014). Qualitative content analysis: A focus on trustworthiness. SAGE Open, 4(1), 2158244014522633. 10.1177/2158244014522633

[nop21976-bib-0010] FirstPost . (2020). International Nurses Day 2020: How nurses are contributing during the COVID‐19 pandemic . Available at: https://www.firstpost.com/health/international‐nurses‐day‐2020‐how‐nurses‐are‐contributing‐during‐the‐covid‐19‐pandemic‐8358231.html

[nop21976-bib-0011] Galvin, J. , Richards, G. , & Smith, A. P. (2020). A longitudinal cohort study investigating inadequate preparation and death and dying in nursing students: IMPLICATIONS for the aftermath of the COVID‐19 pandemic. Frontiers in Psychology, 11, 2206. 10.3389/fpsyg.2020.02206 32982890 PMC7477344

[nop21976-bib-0012] Gharebaghi, R. , Heidary, F. , & Pourezzat, A. A. (2021). Serial deaths of young trainee physicians in Iran during COVID‐19 pandemic; messages to policy makers. Frontiers in Health Services, V2, 777065. 10.3389/frhs.2022.777065 PMC10012792 36925768

[nop21976-bib-0013] Giménez‐Espert, M. D. , Prado‐Gascó, V. , & Soto‐Rubio, A. (2020). Psychosocial risks, work engagement, and job satisfaction of nurses during COVID‐19 pandemic. Frontiers in Public Health, 8, 566896. 10.3389/fpubh.2020.566896 33330313 PMC7716584

[nop21976-bib-0014] Goveas, J. S. , & Shear, M. K. (2021). Grief and the COVID‐19 pandemic in older adults. Focus, 19(3), 374–378. 10.1176/appi.focus.19303 34690607 PMC8475942

[nop21976-bib-0015] Greenberg, N. , Docherty, M. , Gnanapragasam, S. , & Wessely, S. (2020). Managing mental health challenges faced by healthcare workers during covid‐19 pandemic. BMJ, 368, m1211. 10.1136/bmj.m1211 32217624

[nop21976-bib-0016] Hernández‐Fernández, C. , & Meneses‐Falcón, C. (2021). I can't believe they are dead. Death and mourning in the absence of goodbyes during the COVID‐19 pandemic. Health & Social Care in the Community, 30(4), 1220–1232. 10.1111/hsc.13530 PMC8444868 34363273

[nop21976-bib-0017] Kostka, A. M. , Borodzicz, A. , & Krzemińska, S. A. (2021). Feelings and emotions of nurses related to dying and death of patients–a pilot study. Psychology Research and Behavior Management, 4, 705–717. 10.2147/PRBM.S311996 PMC8187100 34113186

[nop21976-bib-0018] Kubler‐Ross, E. (1969). On death and dying. The Macmillan Company.

[nop21976-bib-0019] Lobb, E. A. , Oldham, L. , Vojkovic, S. , Kristjanson, L. J. , Smith, J. , Brown, J. M. , & Dwyer, V. W. (2010). Frontline grief: The workplace support needs of community palliative care nurses after the death of a patient. Journal of Hospice & Palliative Nursing, 12(4), 225–233. 10.1097/NJH.0b013e3181dceadc

[nop21976-bib-0020] Maddrell, A. (2020). Bereavement, grief, and consolation: Emotional‐affective geographies of loss during COVID‐19. Dialogues in Human Geography, 10(2), 107–111. 10.1177/2043820620934947

[nop21976-bib-0021] McCallum, K. J. , Walthall, H. , Aveyard, H. , & Jackson, D. (2021). Grief and nursing: Life and death in the pandemic. Journal of Advanced Nursing, 77(5), 2115–2116. 10.1111/jan.14815 33606276 PMC8014175

[nop21976-bib-0022] McGettigan, D. , Clark, H. , Burgess, L. , Callanan, A. , & Leen, B. (2020). Rapid evidence review: What supports are needed for frontline healthcare staff following the death of a colleague due to COVID‐19? Health Service Executive.

[nop21976-bib-0023] Mohammed, S. , Peter, E. , Killackey, T. , & Maciver, J. (2021). The “nurse as hero” discourse in the COVID‐19 pandemic: A poststructural discourse analysis. International Journal of Nursing Studies, 117, 103887. 10.1016/j.ijnurstu.2021.103887 33556905 PMC9749900

[nop21976-bib-0024] Moore, K. J. , Sampson, E. L. , Kupeli, N. , & Davies, N. (2020). Supporting families in end‐of‐life care and bereavement in the COVID‐19 era. International Psychogeriatrics, 32(10), 1245–1248. 10.1017/S1041610220000745 32349850 PMC7235296

[nop21976-bib-0025] Mortazavi, S. S. , Shahbazi, N. , Taban, M. , Alimohammadi, A. , & Shati, M. (2021). Mourning during corona: A phenomenological study of grief experience among close relatives during COVID‐19 pandemics. OMEGA‐Journal of Death and Dying, 20, 00302228211032736. 10.1177/00302228211032736 34282960

[nop21976-bib-0026] Neimeyer, R. A. (2014). The changing face of grief: Contemporary directions in theory, research, and practice. Progress in Palliative Care, 22(3), 125–130. 10.1179/1743291X13Y.0000000075

[nop21976-bib-0027] Rabow, M. W. , Huang, C. H. , White‐Hammond, G. E. , & Tucker, R. O. (2021). Witnesses and victims both: Healthcare workers and grief in the time of COVID‐19. Journal of Pain and Symptom Management, 62(3), 647–656. 10.1016/j.jpainsymman.2021.01.139 33556494 PMC7864782

[nop21976-bib-0028] Rotter, J. C. (2000). Family grief and mourning. The Family Journal, 8(3), 275–277. 10.1177/1066480700083010

[nop21976-bib-0029] Różyk‐Myrta, A. , Brodziak, A. , & Kołat, E. (2021). Nurses as new heroes of modern times. International Nursing Review, 68(2), 163–165. 10.1111/inr.12676 33761142

[nop21976-bib-0030] Selman, L. E. , Chao, D. , Sowden, R. , Marshall, S. , Chamberlain, C. , & Koffman, J. (2020). Bereavement support on the frontline of COVID‐19: Recommendations for hospital clinicians. Journal of Pain and Symptom Management, 60(2), e81–e86. 10.1016/j.jpainsymman.2020.04.024 32376262 PMC7196538

[nop21976-bib-0031] Shear, M. K. , Simon, N. , Wall, M. , Zisook, S. , Neimeyer, R. , Duan, N. , Reynolds, C. , Lebowitz, B. , Sung, S. , Ghesquiere, A. , & Gorscak, B. (2011). Complicated grief and related bereavement issues for DSM‐5. Depression and Anxiety, 28(2), 103–117. 10.1002/da.20780 21284063 PMC3075805

[nop21976-bib-0032] Shoraka, H. R. , Hashemi, S. A. , Asghari, D. , Chegeni, M. , Arzamani, N. A. , Sadidi, N. , Dousti, Z. , & Kaviyani, F. (2022). Mourning during COVID‐19 pandemic in Bojnurd, a city in northeast of Iran: A qualitative study. Journal of Iranian Medical Council, 5(2), 263–273. 10.18502/jimc.v5i2.10464

[nop21976-bib-0033] Vachon, M. L. (2001). The nurse's role: The world of palliative care nursing. In B. R. Ferrell & N. Coyle (Eds.), Textbook of palliative nursing (pp. 647–662). Oxford University Press.

[nop21976-bib-0034] Voultsos, P. , Tsompanian, A. , Deligianni, M. , & Tsaroucha, A. K. (2022). A qualitative study of nursing practitioners' experiences on dying alone due to COVID‐19 in Greece. Frontiers in Public Health, 10, 981780. 10.21203/rs.3.rs-1345751/v1 36339201 PMC9634155

[nop21976-bib-0035] Wallace, C. L. , Wladkowski, S. P. , Gibson, A. , & White, P. (2020). Grief during the COVID‐19 pandemic: Considerations for palliative care providers. Journal of Pain and Symptom Management, 60(1), e70–e76. 10.1016/j.jpainsymman.2020.04.012 PMC7153515 32298748

[nop21976-bib-0036] World Health Organisation . (2021). Health and care worker deaths during COVID‐19 . https://www.who.int/news/item/20‐10‐2021‐health‐and‐care‐worker‐deaths‐during‐covid‐19

[nop21976-bib-0037] Zhai, Y. , & Du, X. (2020). Loss and grief amidst COVID‐19: A path to adaptation and resilience. Brain, Behavior, and Immunity, 87, 80–81. 10.1016/j.bbi.2020.04.053 32335197 PMC7177068

